# Spontaneous rupture of a mucinous borderline ovarian tumour in late pregnancy: a case report

**DOI:** 10.3389/fonc.2026.1954702

**Published:** 2026-09-15

**Authors:** Xinjun Li, Yanan Ren, Shuo Xu, Xiangnan Zhang, Kaiyun Qin, Liyun Song, Shengpu Wang

**Affiliations:** 1 Department of Gynecology, Hebei General Hospital, Shijiazhuang, China; 2 Department of Pathology, Hebei General Hospital, Shijiazhuang, China; 3 Department of Obstetrics, The Second Hospital of Hebei Medical University, Shijiazhuang, China

**Keywords:** case report, fertility-sparing surgery, mucinous borderline ovarian tumour, multidisciplinary management, ovarian tumour rupture, pregnancy

## Abstract

**Background:**

Mucinous borderline ovarian tumours (mBOTs) during pregnancy are rare, and spontaneous rupture in the third trimester is a life-threatening emergency. Most adnexal masses with stable imaging are managed conservatively, which risks delayed intervention. The optimal surveillance and surgical strategies for mBOTs in late pregnancy remain unclear.

**Case description:**

A 26-year-old primigravida with a 10-year history of an adnexal cyst presented at 29 + 1 weeks with acute abdominal pain. Serial ultrasounds documented tumour growth from 4.0 cm pre-pregnancy to 17.46 cm (Figure 1), with morphological deterioration. Emergency laboratory tests revealed elevated AFP, CA19-9, and HE4 levels, which, combined with imaging findings, raised suspicion of a neoplastic process. Emergency laparotomy at 29 + 1 weeks revealed a ruptured right ovarian tumour with 1000 mL of mucinous ascites. Frozen section suggested a borderline tumour. Fertility-sparing staging surgery (adnexectomy, peritoneal biopsies, omentectomy) was performed. Postoperative pathology, confirmed by expert consultation, diagnosed a mBOT (atypical proliferative mucinous tumour) with 85% borderline components, Ki-67 ~30%, and no intraepithelial carcinoma, no microinvasion, and no invasive carcinoma The patient recovered uneventfully and she had a spontaneous vaginal delivery at 39 + 2 weeks of gestation, delivering a healthy full-term male infant (2, 840 g, Apgar 10/10). Postpartum imaging and tumour markers showed no evidence of residual disease.

**Conclusion:**

This case suggests that conventional expectant management may be inadequate for pre-pregnancy cystic adnexal masses with growth trajectory, highlights the importance of multidisciplinary evaluation and stratified monitoring, and illustrates that emergency fertility-sparing staging surgery in the third trimester is feasible and achieved favourable maternal and neonatal outcomes in a patient with a ruptured mucinous borderline tumour, while preserving fertility.

## 1 Introduction

Borderline ovarian tumours (BOTs) are neoplasms of low malignant potential, accounting for 15%–20% of epithelial ovarian malignancies with an incidence of 1.8–4.8 per 100, 000 women annually (1). Mucinous BOTs (mBOTs) also referred to as atypical proliferative mucinous tumours, are less common than serous subtypes and typically present as multilocular cystic lesions with indolent progression (2).

Most gestational ovarian masses are small, asymptomatic, and managed with expectant observation (3). Ovarian torsion is the most common complication of adnexal masses during pregnancy, whereas spontaneous rupture of large mBOTs in late pregnancy is extremely rare, with only isolated case reports documented in the literature (4–6). Tumour rupture with intraabdominal leakage of mucinous contents can induce peritonitis and threaten maternal–fetal safety (7).

Unified surveillance guidelines for pre-existing adnexal masses during pregnancy remain absent (3, 8). Routine ultrasonography without dynamic tumour marker monitoring or multidisciplinary evaluation may miss high-risk progression (9). Emergency staging surgery in late pregnancy is technically challenging due to uterine enlargement and fetal risks (10). We report a case of spontaneous rupture of a large mBOT in the third trimester, discuss clinical pitfalls in surveillance, surgical decision-making, and long-term follow-up management. Published descriptions of mBOTs with mid-trimester accelerated growth followed by spontaneous rupture in late pregnancy remain scarce. We report this unique case to summarize surveillance deficiencies and share multidisciplinary oncologic management experience.

## 2 Case report

### 2.1 Clinical history and chief complaint

A 26-year-old primigravida (G1P0) at 29 + 1 weeks of gestation presented to the gynecology department with progressive right upper abdominal pain, nausea, and hiccups lasting four hours after defecation. She denied vaginal bleeding, fluid leakage, contractions, fever, vomiting, or diarrhea.

The patient had a more than 10-year history of a right adnexal cyst with regular conservative follow-up. Pre-pregnancy ultrasonography demonstrated a 4.0 cm right adnexal cyst, initially suspected to be an endometriotic cyst; serum CA125 was reportedly elevated on one occasion (exact value unavailable). Conception was spontaneous; no assisted reproductive technologies were used. No systematic tumour marker monitoring was performed during the 10-year period before pregnancy. The patient had undergone a transabdominal appendectomy for acute appendicitis at the age of 13 at an outside institution; the archived pathological slides were unavailable for review. She had no prior pelvic surgery, pelvic inflammatory disease, or family history of malignant tumours.

### 2.2 Serial prenatal ultrasonography and tumour growth trajectory

Pre-pregnancy ultrasound demonstrated a 4.0 cm unilocular cyst with no solid components and no hyperechogenicity (IOTA B1 feature).

Serial prenatal ultrasounds documented continuous enlargement and morphological deterioration throughout pregnancy (**Figure 1**). The tumour enlarged from 4.0 cm pre-pregnancy to 17.46 cm at 29 + 1 weeks, with a steep acceleration after 20 weeks.

**Figure 1:**
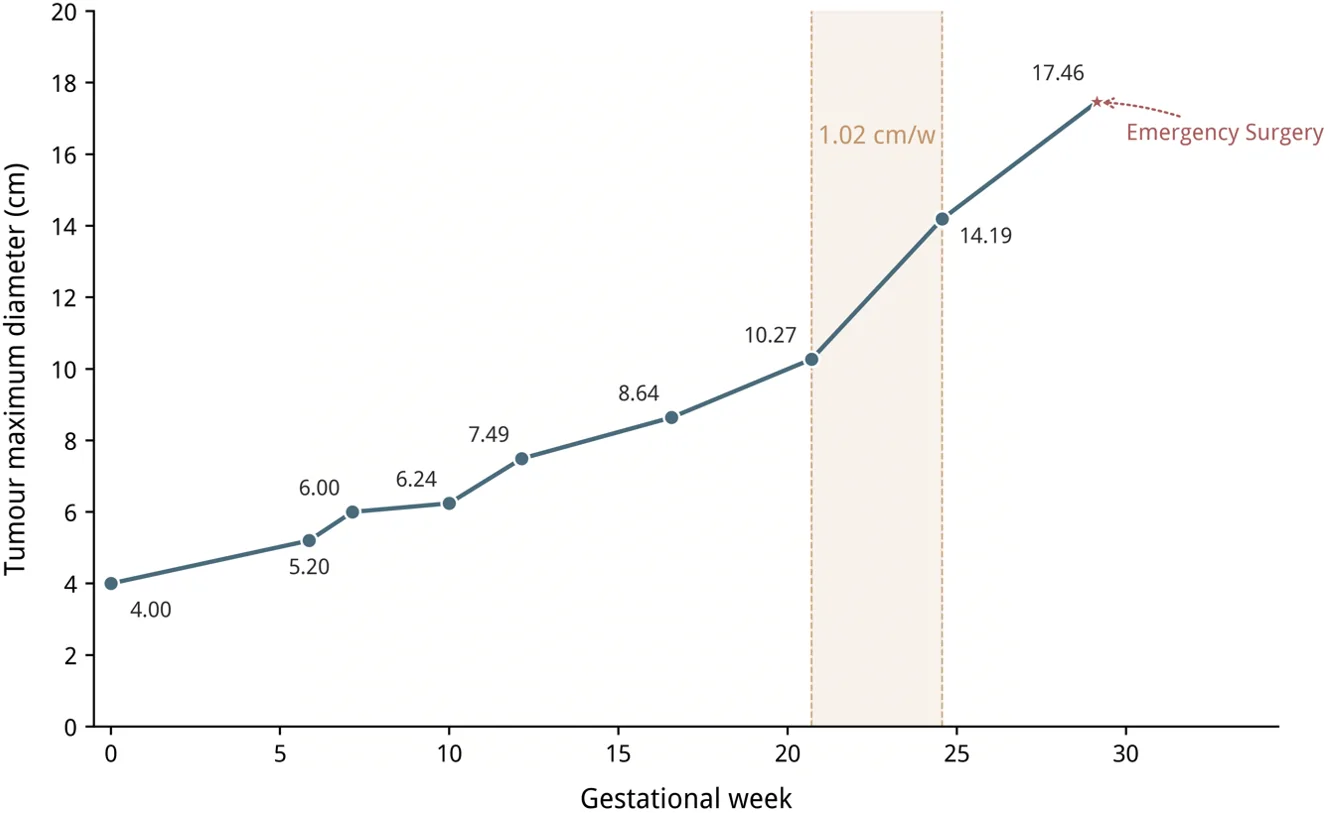
The tumour enlarged from 4.0 cm pre-pregnancy to 17.46 cm at 29 + 1 weeks of gestation on a linear gestational age scale. The growth rate peaked between 20 + 5 and 24 + 4 weeks (1.02 cm/w), compared with 0.39 cm/week in the preceding interval (16 + 4 to 20 + 5 weeks) and 0.72 cm/week thereafter (24 + 4 to 29 + 1 weeks). The orange shaded area denotes the accelerated growth phase; the asterisk indicates the time of emergency surgery with pre-operative tumour measurement.

Using IOTA terminology, the lesion evolved as follows: at 5w6d, it was a unilocular cyst (5.2 cm) without solid components (B1); at 7w1d, a unilocular cyst (6.0 cm, B1); at 10w, a multilocular cyst (6.24 cm) with 3 locules and thin septa (B2); at 12w1d, a multilocular cyst (7.49 cm, B2); at 16w4d, a multilocular cyst (8.64 cm, B2); at 20w5d, a multilocular-solid cyst with more than 10 locules and papillary projections (10.27 cm) — this was the point at which the lesion should have been reclassified as suspicious under IOTA Simple Rules (M1 feature); at 24w4d, the multilocular-solid cyst with multiple solid components had increased to 14.19 cm, with colour score 3; and at 29 + 1 weeks (admission), the mass measured 17.46 cm and showed multiple thick septa (>3 mm), multiple solid components, and colour score 4, with free fluid. Free pelvic fluid was detected on the final prenatal ultrasound at 29 + 1 weeks, confirming rupture.

Only a single CA125 measurement was obtained at 6 weeks of gestation (77.08 U/mL), without serial dynamic tumour marker monitoring during pregnancy.

### 2.3 Physical examination and laboratory findings

On admission, vital signs were: temperature 37.3 °C, heart rate 114 bpm, respiratory rate 21/min, and blood pressure 134/72 mmHg. Physical examination revealed a gravid abdomen with diffuse abdominal tenderness, most prominent in the right upper quadrant. The fetal heart rate was 152 bpm.

Emergency laboratory tests showed marked elevations of tumour markers: AFP 205.995 ng/mL, CA19-9 138.372 U/mL, and HE4 254.908 pmol/L (**Table 1**), suggesting a high-risk epithelial ovarian tumour. CA125 was within normal limits (14.858 U/mL; reference < 35 U/mL). Emergency pelvic ultrasound identified a huge right adnexal cystic-solid mass measuring 174 mm × 153 mm × 113 mm with multiple septations, floating flocculent echoes, and abundant pelvic free fluid with a maximum depth of 83 mm in the right lower abdomen (**Figure 2A**).

**Table 1:** Laboratory findings on admission.

Parameter	6w	29 + 1w	2w post-op	1w pp	Non-pregnant ref.
AFP	—	**205.995**	**152.643**	**71.284**	< 10 ng/mL
CA125	77.08	14.858	20.137	16.368	< 35 U/mL
CA19-9	—	**138.372**	14.580	4.820	< 35 U/mL
HE4	—	**254.908**	81.273	74.810	< 140 pmol/L

Bold: above normal reference range. —: not measured.

6w: 6 weeks gestation; 29 + 1w: 29 + 1 weeks gestation (admission); 2w post-op: 2 weeks after surgery; 1w pp: 1 week postpartum. Non-pregnant ref.: non-pregnant reference range.

**Figure 2:**
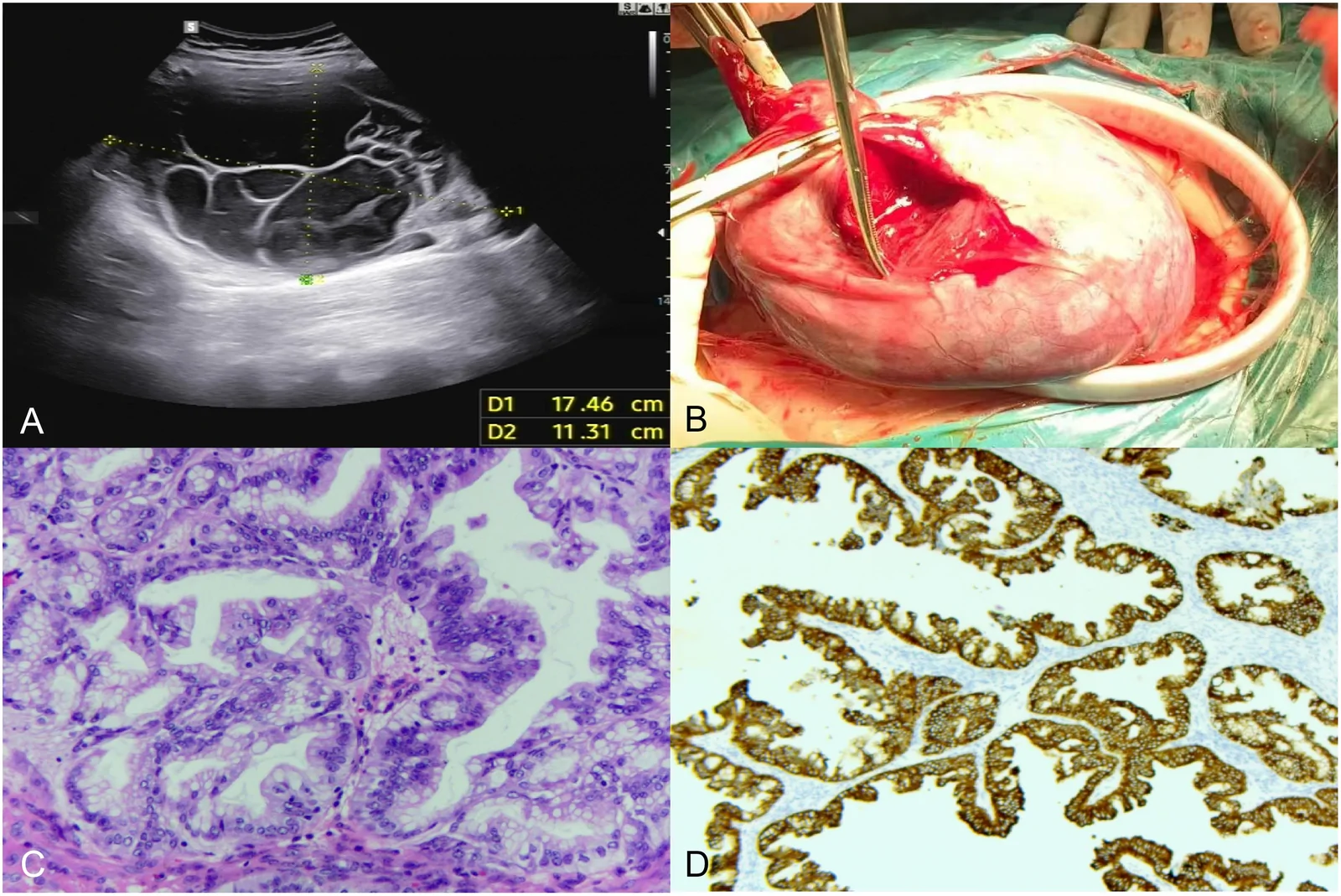
Preoperative ultrasound, intraoperative findings, and pathological features of the tumour. **(A)** Preoperative ultrasound showing a large cystic-solid mass with multiple septations and flocculent echoes. **(B)** Intraoperative photograph demonstrating the ruptured right ovarian tumour with mucinous content spillage. **(C)** Hematoxylin and eosin staining showing a borderline mucinous tumour with complex architecture and cytological atypia (original magnification ×200). **(D)** Immunohistochemical staining for CK7 showing diffuse cytoplasmic positivity (×200).

### 2.4 Perioperative management and surgery

The patient presented with acute severe abdominal pain and was admitted emergently. After thorough communication with the patient and her family regarding the surgical risks, a multidisciplinary team (MDT) including obstetricians, gynecologic oncologists, anesthesiologists, and neonatologists was immediately convened. Considering the persistent abdominal pain, inflammatory reaction, and confirmed tumour rupture, emergency exploratory laparotomy was performed at 29 + 1 weeks of gestation.

The operation was performed under general anaesthesia, with the patient maintained in a 15-degree left-tilted position to relieve aortocaval compression and optimize uteroplacental perfusion. A 15-cm midline vertical abdominal incision was made to provide adequate surgical exposure. Extremely gentle manipulation was strictly implemented throughout the procedure, and the gravid uterus was carefully protected to avoid repeated serosal irritation, thereby reducing the risk of intraoperative uterine contractions and preterm labour.

Intraoperative findings: Approximately 1000 mL of yellow mucinous ascites was present within the peritoneal cavity. The right ovary was markedly enlarged (16 × 10 × 9 cm) with a 2 cm spontaneous rupture site. The tumour presented a multilocular structure containing viscous mucinous fluid and partial solid components (**Figure 2B**). Peritoneal washings and ascitic fluid were collected for cytological examination. The uterine size corresponded to 29 weeks of gestation. The left adnexa, uterine surface, omentum, intestinal serosa, and diaphragmatic peritoneum were grossly unremarkable. The appendix was absent.

Intraoperative frozen section pathology indicated a borderline mucinous ovarian tumour. Given the patient’s young age (26 years) and strong fertility preservation intention, fertility-sparing surgical staging was performed, including right adnexectomy, multi-site peritoneal biopsy (paracolic gutter, vesicouterine peritoneum, and diaphragmatic peritoneum), and omentectomy. Thorough peritoneal lavage with warm distilled water and normal saline was performed to eliminate residual mucinous tumour contents. A subcutaneous drainage tube was placed routinely. The total operative duration was 90 minutes, with an estimated blood loss of approximately 30 mL, and no intraoperative blood transfusion was required.

Perioperative maternal–fetal management: Magnesium sulfate was continuously infused intravenously for 72 hours for fetal neuroprotection. Intramuscular dexamethasone 5 mg every 12 hours for four doses was administered to promote fetal lung maturation. Electronic fetal heart rate monitoring was performed before and after surgery. Given tumour rupture and intraperitoneal spillage of cyst contents, prophylactic cefazolin 2 g was intravenously administered 30 minutes before skin incision and discontinued within 24 hours postoperatively in the absence of pelvic infection. For venous thromboembolism prophylaxis, mechanical thromboprophylaxis with bilateral sequential compression devices was applied continuously during and after surgery.

### 2.5 Pathological findings

Pathological sampling: The tumour was sampled according to the standard protocol for mucinous ovarian neoplasms, with at least one section per 1–2 cm of maximum tumour diameter. A total of 46 blocks were examined, with additional sections taken from solid areas, septations, and the rupture site.

Postoperative paraffin pathology: Revealed complex local structural changes and significant cellular atypia with suspected malignant transformation (**Figure 2C**).

Expert consultation at Peking University Third Hospital: Confirmed a mucinous borderline tumour (atypical proliferative mucinous tumour) with 85% borderline components, 15% benign mucinous cystadenomatous epithelium, no invasive adenocarcinoma characteristics, and no intraepithelial carcinoma and no microinvasion, but significant proliferative activity (Ki-67 ~30%).

Peritoneal washings and ascitic fluid cytology: The results showed abundant acellular mucin with no malignant cells identified. No features of pseudomyxoma peritonei were present.

Immunohistochemistry: CK7 (+) (**Figure 2D**), partial CK20 (+), Villin (+), CDX-2 (-), SATB2 (-). p53: null pattern.

The immunohistochemical findings should be interpreted in the context of differentiating a primary ovarian mucinous tumour from a metastatic gastrointestinal or appendiceal origin. The immunohistochemical profile (CK7+, partial CK20+, Villin+, CDX2–, SATB2–) is supportive of a primary ovarian mucinous tumour. CK7 positivity argues against a lower gastrointestinal primary, as such metastases are typically CK7– (11). Partial CK20+ is not contradictory—the CK7+/CK20+ pattern is the most common immunophenotype in primary ovarian mucinous tumours (74%) (12). Villin+ reflects intestinal differentiation but has limited specificity (21.4%) and cannot reliably distinguish ovarian from gastrointestinal origin (13). The key finding is CDX2–/SATB2–: SATB2 is highly specific for colorectal/appendiceal origin (97.5% specificity for appendiceal origin) (14), and the CK7+/SATB2– combination distinguishes primary ovarian mucinous tumours from lower gastrointestinal metastases with >95% accuracy (11). Taken together, these findings are most consistent with a primary ovarian mucinous tumour. While the patient had a remote history of appendectomy at the age of 13 at an outside institution, the archived pathological slides were unavailable for review. However, the combination of intraoperative findings and the immunohistochemical profile makes an appendiceal primary unlikely.

### 2.6 Postoperative course and follow-up

Based on these findings, the FIGO 2014 stage was IC2 (capsule ruptured before surgery, negative peritoneal cytology). According to the current NCCN Guidelines (Version 4.2026) (15), observation is the preferred strategy for borderline ovarian tumours without invasive implants, as postoperative adjuvant chemotherapy has not been shown to provide a demonstrated benefit; therefore, it was withheld.

The patient recovered uneventfully, and weekly antenatal care was unremarkable. At 2 weeks postoperatively, tumour markers showed AFP 152.643 ng/mL (slight decline from the pre-operative level), with CA125, CA19-9, and HE4 all within normal limits (**Table 1**). Weekly pelvic ultrasound revealed no tumour recurrence.

At 39 + 2 weeks, she had a spontaneous vaginal delivery of a healthy male infant weighing 2, 840 g (Apgar 10/10). At 1 week postpartum, AFP had declined further to 71.284 ng/mL, with all other markers remaining normal (**Table 1**). Contrast-enhanced CT of the chest, abdomen, and pelvis showed no residual disease. At the time of manuscript submission, the patient was 2 weeks postpartum and remains under regular surveillance.

## 3 Discussion

We report a case of spontaneous rupture of a mBOT in the third trimester with favorable outcomes. The discussion focuses on four aspects: pre-pregnancy diagnostic pitfalls, gestational accelerated growth and tumour marker interpretation, surgical strategy, and postoperative management.

### 3.1 Pre-pregnancy diagnostic pitfalls

Long-term asymptomatic ovarian cysts in reproductive-aged women are often considered benign. Most BOTs are diagnosed at FIGO stage I, with a 10-year survival rate exceeding 95% (16). However, mBOT pathology remains challenging (2).

In this case, the adnexal lesion persisted for more than 10 years and was documented as a 4.0 cm cystic mass four months before conception. Yet neither systematic tumour marker evaluation nor specialized gynecologic oncology consultation was performed before pregnancy. This suggests that long-standing cystic adnexal masses may acquire latent proliferative potential during pregnancy, warranting close monitoring and stratified assessment both before and during pregnancy (8, 17).

### 3.2 Gestational accelerated growth, rupture, and missed surgical opportunity

BOTs may exhibit accelerated growth during pregnancy, possibly influenced by gestational changes. A retrospective study of 17 pregnant BOT patients reported that the most common presenting symptoms were cysts, cyst enlargement, or cyst rupture. Notably, 11 of 17 (64.7%) patients were diagnosed in the third trimester, and 3 cases presented with cyst rupture, suggesting that the risk of acute complications, including rupture, may increase with advancing gestation (5). This pattern is consistent with the clinical course observed in our patient.

In this patient, the tumour increased from 4.0 cm to 17.46 cm at 29 weeks (**Figure 1**), with a steep acceleration after 20 weeks. The window for elective surgical intervention was missed. By 20 weeks, the tumour had already exceeded 8 cm with cystic-solid components. These high-risk features should have prompted intervention. According to ACOG and SOGC guidelines, non-emergent adnexal surgery during pregnancy is ideally performed between 14 and 22 weeks of gestation to minimize maternal and fetal risks (3, 7). However, this opportunity was not taken. The tumour continued to grow and ruptured at 29 weeks. This case highlights a failure in risk stratification. Serial imaging findings and elevated tumour markers were not synthesized to trigger timely multidisciplinary reassessment.

### 3.3 Role of a multiparameter tumour marker panel in differential diagnosis

Tumour markers have limited diagnostic value for adnexal masses in pregnancy; their principal role is in post-operative surveillance. CA125 can be physiologically elevated in early pregnancy and is often lower in BOTs than in invasive cancers. HE4 is decreased in normal pregnancy (18, 19), making the marked elevation in this case noteworthy. CA19–9 is commonly elevated across the benign-borderline-malignant spectrum of mucinous tumours and does not reliably predict subtype (20). AFP rises physiologically during gestation, with third-trimester levels up to 250 ng/mL (21).

In our patient, AFP (205.995 ng/mL), CA19-9 (138.372 U/mL), and HE4 (254.908 pmol/L) were synchronously elevated at admission, while CA125 was normal. AFP fell at the upper limit of the gestational range, whereas CA19–9 and HE4—despite pregnancy-related physiological changes—were markedly elevated. These findings raised clinical vigilance, but none can be interpreted as definitive evidence of malignancy in the gestational context. Notably, although AFP is classically associated with ovarian germ cell tumours, sporadic cases of mucinous ovarian tumours with elevated serum AFP have been documented in non-pregnant patients (22). Nevertheless, we cannot definitively confirm tumour-derived AFP secretion, as maternal AFP physiologically rises in late pregnancy. The markers were obtained after rupture and played an adjunctive role; the primary actionable information came from serial imaging. This case supports using tumour markers as an adjunct to imaging-based surveillance, with their principal value in post-operative follow-up.

### 3.4 Surgical strategy and timing

Fertility-sparing surgery is preferred for stage I BOT patients desiring fertility preservation (23–26). A retrospective analysis of 313 stage I BOT patients showed recurrence rates of 30.3% for cystectomy, 11% for unilateral adnexectomy, and 1.7% for bilateral adnexectomy (p < 0.001); for the mucinous subtype specifically, rates were 17%, 13%, and 4%, respectively (25).

The conventional elective surgical window for benign gestational masses is 14–22 weeks of gestation (3, 7). However, this principle does not apply to tumours with progressive growth, morphological deterioration, and elevated markers. Rupture with massive ascites and peritonitis at 29 weeks constituted an absolute emergency indication regardless of gestational age (7).

### 3.5 Challenges in pathological diagnosis of mBOTs

The pathological diagnosis of mucinous borderline ovarian tumours (mBOTs) is inherently challenging due to their heterogeneous morphology and the subjective nature of interpreting stromal invasion. Interobserver variability in distinguishing mBOT from mucinous carcinoma is well documented, with only moderate agreement even among expert gynaecological pathologists (26).

This diagnostic complexity is illustrated in our case. The intraoperative frozen section diagnosis was concordant with the final expert consultation at Peking University Third Hospital: both confirmed a mucinous borderline tumour without invasive features. The discordance arose between the initial local paraffin interpretation — which raised concern for “suspected malignant transformation” — and the expert review, which confirmed a mucinous borderline tumour with high proliferative activity (Ki-67 ~30%) but no intraepithelial carcinoma, no microinvasion, and no invasive carcinoma. This discrepancy reflects interobserver variability in paraffin-section interpretation of mucinous neoplasms (27).

Comprehensive pathological sampling (46 blocks from a 17.46 cm tumour, exceeding the recommended one block per 1–2 cm of maximum tumour diameter) and expert review were essential to exclude an invasive component. Accurate pathological stratification via senior expert consultation was the critical basis for our clinical decision against adjuvant chemotherapy.

The p53 finding, although unrelated to the diagnostic discordance, warrants brief comment. Although aberrant p53 expression is uncommon in mucinous borderline tumours, it has been documented in a subset of cases (28). Importantly, p53 expression status in mucinous borderline tumours lacks prognostic significance (29) and does not alter the diagnosis or clinical management when no invasive features are identified.

### 3.6 Postoperative management and long-term surveillance

Adjuvant chemotherapy has no definite clinical benefit for BOT patients. BOTs are characterized by low proliferative activity and insensitivity to cytotoxic chemotherapy (9). Even in patients with microinvasive implantation, the value of adjuvant chemotherapy remains controversial (30). Postoperative recurrence rates range from 5% to 34%; most recurrent lesions remain borderline and rarely progress to invasive carcinoma, and recurrent tumours can usually be managed successfully with reoperation (30, 31).

Given three high-risk factors in this case—tumour rupture, massive intraperitoneal mucinous fluid, and elevated Ki-67 index—we established an individualized stratified long-term surveillance protocol: postpartum contrast-enhanced CT to exclude occult implants, serial monitoring of AFP, CA19-9, and HE4 combined with annual pelvic ultrasonography. Tumour rupture has been statistically confirmed as an independent risk factor for BOT recurrence (32), requiring intensified surveillance. Given the delayed median recurrence time in BOTs, a minimum of 10 years of follow-up or lifelong surveillance is warranted to minimize long-term recurrence risks (33).

### 3.7 Take-away lessons

Three key lessons emerge from this case. First, long-standing pre-pregnancy adnexal cystic masses warrant pre-conception gynecologic oncology evaluation and close monitoring during pregnancy. Second, tumour rupture in late pregnancy—even at 29 weeks—does not preclude fertility-sparing surgery; under multidisciplinary support, emergency oncological staging is feasible and can achieve favorable maternal-fetal outcomes. Third, given the inherent diagnostic challenges of mucinous borderline tumours, standardised pathological sampling and multidisciplinary pathology review—including consultation with reference centres—are essential to achieve accurate diagnosis and appropriate risk stratification.

### 3.8 Study limitations

This study has several limitations. First, as a single-case retrospective report without control cohorts and statistical analysis, direct causality between gestational hormonal stimulation and tumour borderline transformation and progression cannot be definitively established. Second, pelvic MRI was not performed during pregnancy, lacking multi-dimensional imaging evidence for evaluating subtle tumour infiltration and occult pelvic lesions. Third, the postoperative follow-up duration is relatively limited, and the long-term recurrence patterns and prognosis of mBOTs require continued observation and verification.

## 4 Patient perspective

The patient shared her experience: “I had known about the ovarian cyst for over 10 years and was told it was nothing to worry about. When I became pregnant, the doctors said it was likely just an endometrioma and advised me to have regular check-ups. I was completely unprepared when sudden severe pain struck at 29 weeks. At that moment, I feared not only for my own life but also for my baby’s. The doctors explained that emergency surgery was necessary, which was terrifying because I was afraid of losing the pregnancy. I am deeply grateful to the multidisciplinary team at Hebei General Hospital for saving both me and my baby. The consultation with Peking University Third Hospital gave us confidence in the diagnosis, and the decision to avoid chemotherapy during pregnancy helped me carry the baby to full term. I hope that sharing my case can help other women with similar conditions receive better care and avoid what I went through.”

## Data Availability

The original contributions presented in the study are included in the article/supplementary material. Further inquiries can be directed to the corresponding author.
